# Engineering of bespoke photosensitiser–microbe interfaces for enhanced semi-artificial photosynthesis

**DOI:** 10.1039/d4sc00864b

**Published:** 2024-05-21

**Authors:** Imogen L. Bishara Robertson, Huijie Zhang, Erwin Reisner, Julea N. Butt, Lars J. C. Jeuken

**Affiliations:** a Leiden Institute of Chemistry, Leiden University PO Box 9502 Leiden 2300 RA the Netherlands; b Yusuf Hamied Department of Chemistry, University of Cambridge Cambridge CB2 1EW UK; c School of Chemistry and School of Biological Sciences, University of East Anglia Norwich Research Park Norwich NR4 7TJ UK

## Abstract

Biohybrid systems for solar fuel production integrate artificial light-harvesting materials with biological catalysts such as microbes. In this perspective, we discuss the rational design of the abiotic–biotic interface in biohybrid systems by reviewing microbes and synthetic light-harvesting materials, as well as presenting various approaches to coupling these two components together. To maximise performance and scalability of such semi-artificial systems, we emphasise that the interfacial design requires consideration of two important aspects: attachment and electron transfer. It is our perspective that rational design of this photosensitiser–microbe interface is required for scalable solar fuel production. The design and assembly of a biohybrid with a well-defined electron transfer pathway allows mechanistic characterisation and optimisation for maximum efficiency. Introduction of additional catalysts to the system can close the redox cycle, omitting the need for sacrificial electron donors. Studies that electronically couple light-harvesters to well-defined biological entities, such as emerging photosensitiser–enzyme hybrids, provide valuable knowledge for the strategic design of whole-cell biohybrids. Exploring the interactions between light-harvesters and redox proteins can guide coupling strategies when translated into larger, more complex microbial systems.

## Abstract 2

To maximise performance and scalability of biohybrid systems for solar fuel generation, we emphasise the need for rational design of the biotic–abiotic interface, taking into consideration two important aspects: attachment and electron transfer.

## Graphical abstract

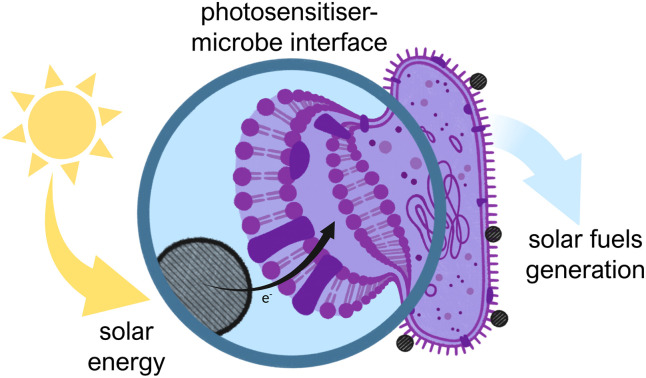

## Introduction

Generating fuels from renewable energy, such as sunlight, is of paramount importance in our urgent transition away from fossil fuels. In its most basic form, three components are required for photoredox catalysis: a catalyst for chemical reduction, a catalyst for chemical oxidation, and a light absorber to harvest solar energy. These components can be synthetic, natural, or a combination of both. Semi-artificial photosynthesis has been previously defined as interfacing biological catalysts, which provide unparalleled catalytic specificity for the production of desirable chemicals, with synthetic light-harvesting materials to create biohybrid systems.^1^ Here, we focus specifically on the integration of microbial catalysts directly with synthetic nanoparticle light-harvesters. We refer the reader to previous reviews on other systems of semi-artificial photocatalysis, including photoelectrochemical cells (PECs), which are not discussed here.^2,3^ While the use of microbes as biological catalysts in solution with light-harvesting nanoparticles has been investigated for decades,^4^ a landmark study in 2016 initiated a renaissance of research into these biotechnologies by demonstrating that nanoparticles could be synthesised and then localised at the surface of the microbe.^5^ A long-lived physical interface between microbe and photosensitiser eliminates diffusion as a limiting factor. Synthetic materials can have advantages over natural photosystems in terms of resistance to photodamage; some subunits of natural photosystems require repair as often as every 30 minutes.^6^ Further, microbial approaches exploit the capability of live cells to self-replicate and repair, reducing the input of materials such as purified proteins that require costly and time-consuming production. The ability to genetically program cells also provides opportunities to engineer them as factories for a specific chemical product.^7,8^

While light-harvesters and microbes have been previously reviewed in depth, and whole-cell biohybrids discussed and analysed,^2,9–16^ the challenge of creating a defined electron transfer interface between photosensitiser and microbe has only been highlighted.^17^ The nature of this abiotic–biotic interface in existing biohybrids is often unclear and difficult to characterise retrospectively.^18^ Thus, the question we aim to address here is: how can we establish controllable and characterisable pathways for electron transfer, from photosensitiser to cytoplasmic enzymes, that can be built-in by design? What research should we turn to for creating these biohybrid blueprints, and where are remaining gaps of knowledge that prevent progression? In this perspective, we propose that the rational design of these systems should exploit microbes with natural mechanisms of electron uptake, and involve engineered attachment of the photosensitiser to the biological catalyst. We support this view with a detailed discussion of research that we hope can benefit the ongoing optimisation of whole-cell biohybrids in the future.

## Components for semi-artificial photosynthesis

**Fig. 1:**
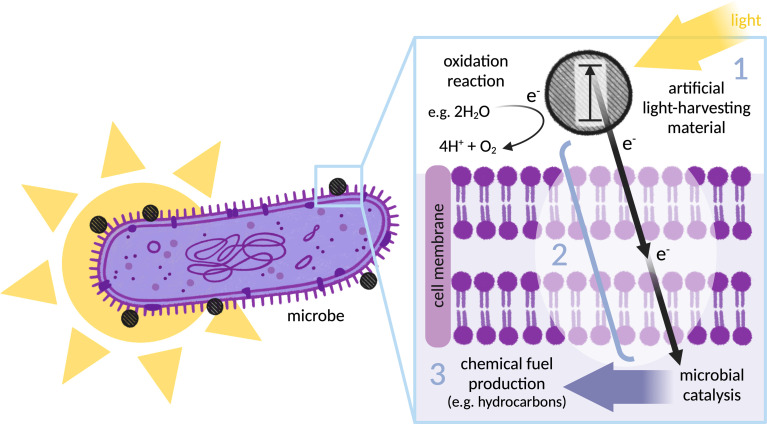
Illustration of the biohybrid concept (in this case using the microbe as a site of reductive catalysis and fuel production): an illuminated (Gram-negative) bacterium, with an inset showing a light-harvesting nanoparticle located on the outer membrane. (1) Artificial light-harvesting material (grey dashed circle) transitions to excited state on illumination: schematically indicated is an electron (e^−^) that moves into a higher energy orbital (in a molecular photosensitiser) or from the valence band to the conduction band (in a nanoparticle). (2) Electron transfer between the light-harvester and the site of catalysis: photoelectrons must be generated in close proximity to the microbe before passing through the microbial membrane to drive intracellular catalysis. (3) Microbial catalysis: the organism is selected or engineered to express enzymes that catalyse production of a desired chemical product. Finally, the light-harvester must be replenished by an oxidation reaction to complete the redox cycle, here indicated by catalytic water oxidation.

Construction of a controllable, optimisable and scalable biohybrid requires consideration of three important areas: selection of an effective light absorber for photoelectron generation (together with a catalyst for regeneration and preferably the synthesis of an added-value chemical); selection of a suitable microbe for catalysis; and finally, electronic coupling of the two *via* a defined electron transfer pathway, from (photo)electron generation through to intracellular catalysis (see Fig. 1). Most research has focused on using the microbe as a site of fuel production through reductive catalysis, although some recent studies reversed this concept and instead use the microbe as an electron donor.^19^ The components discussed in this review are broadly applicable to both designs.

A wide range of artificial light-harvesters have been applied, including organic and inorganic dyes, nanomaterials, and organic semiconductors.^12,20,21^ It is essential to consider the compatibility of these materials with the microbes involved in each system, along with characteristics such as band gap, redox potential, excited state lifetime, stability, and ease of chemical modification. Designing an interaction between the light-harvester and microbe will rely heavily on the mode by which electrons are able to be internalised. Several successful biohybrid systems for fuel production have not fully characterised their photosensitiser–microbe interface for electron transfer, limiting both controllability and optimisability. Other biohybrid systems make use of redox mediators.^22–26^ Mediators are membrane soluble electron carrier molecules that can shuttle electrons between the extracellular environment and the cytoplasm. While this concept works in systems that involve a sacrificial electron donor (SED) to regenerate the light-harvesting material and is valuable at the research stage, systems addressing the ultimate vision of catalysing an oxidative half reaction with a reactant other than a SED could be short-circuited in the presence of redox mediator. Separating the oxidative and reductive half-reaction in different compartments with macroscopic (photo)electrodes would present a potential solution to this short-circuiting. However, there are several disadvantages to these assemblies of macroscopic wiring: they are technically more complicated, and rely on electrodes and ion-exchange membranes which can be both toxic and expensive. Maintaining the neutral pH required by biohybrids is difficult in a set-up where mass transport can create large pH gradients at the electrode interface. Moreover, the single compartment aqueous systems discussed in this perspective are intrinsically compatible with tubular photobioreactors,^27^ making scalable solar light harvesting relatively easy. For a self-generating system, it is also important to consider that growing microorganisms is more controllable in solution than on electrodes.

The case will be presented for exploiting the defined, naturally evolved, characterisable pathways of transmembrane electron transfer in exoelectrogenic bacteria as a simple method for electronically coupling extracellular photoelectrons with intracellular enzymes. Emerging molecular details of these pathways can provide context for informed advances. We argue here that rational design of a specific, engineered interaction at the abiotic–biotic interface is not only superior to interaction by diffusion but also fundamental for systems to be controllable, optimisable and scalable. This perspective presents a range of strategies used to couple photosensitisers with proteins, a discussion that aims to assist in designing the conjugation of photosensitisers to biological components at the surface of microbes.

## Current approaches in biohybrid research

Current biohybrid systems can be classified broadly into two themes: (1) synthesised nanomaterials can be directly added to the growth-media of the bacteria and (2) nanoparticles can be bio-precipitated by the bacteria itself upon addition of starting materials. Furthermore and as mentioned above, a distinction can be made on whether or not redox mediators are used to shuttle electrons from the nanomaterial to the intracellular environment of the microbe. Each of these approaches has a distinct effect on the photocatalytic performance (refer to Table 1 for a summary of key features of the systems discussed, including performance parameters).

**Table 1:** Properties of selected biohybrid systems

Photosensitiser		Microbe			Physical attachment	Electron transfer (ET) interface	System performance			Ref.
Material	Toxicity	Species	Gram *+/−*	Reaction			Quantum efficiency (QE)/quantum yield (QY)	Product yield	Light source	
OF/PTP	N/A	*R. palustris*	−	H_2_ production	None	MV mediated ET from light-harvester to microbe	N/A	∼1.25 μmol H_2_ produced in 2 h (20 μM OF, 20 μM PTP, 0.5 mM MV, OD_660_ = 1.0)	Visible light, 100 W m^−2^	22
TiO_2_ NPs	N/A	Recombinant *E. coli*	−	H_2_ production	None	MV mediated ET from light-harvester to microbe	Apparent QY: 31.2% (350 nm)	0.95 mmol H_2_ produced in 2 h (50 mg semiconductor, 5 mM MV, 100 mg wet cells)	Xenon lamp, 330 W	23, 24
Eosin Y/Ru(bpy)_3_^2+^	N/A	*S. oneidensis*	−	H_2_ production/CO_2_ fixation	None	MV mediated ET from light-harvester to microbe	Apparent QE (H_2_ production): 0.6 ± 0.1% (Eosin Y, 500 nm)/0.5 ± 0.1% (Ru(bpy)_3_^2+^, 450 nm)	H_2_ produced in 40 min: ∼0.17 μmol (Eosin Y)/∼15 nmol (Ru(bpy)_3_^2+^); (0.11 mM photosensitiser, 0.3 mM MV, OD_590_ = ∼0.25)	Visible light, 700 W m^−2^	25
Eosin Y/Ru(bpy)_3_^2+^	N/A	Recombinant *E. coli*	−	H_2_ production	None	MV mediated ET from light-harvester to microbe	Apparent QY: 4.8% (Eosin Y, DQ mediator)	TON (μmol H_2_ mL^−1^ OD_600_^−1^): 10.2 ± 0.7 (Eosin Y, in 184 h)/6.2 ± 1.0 (Ru(bpy)_3_^2+^, in 360 h); (100 μM photosensitiser, 1 mM MV, OD_600_ = 5)	White light, 4000 lx	26
CdS NPs	N/A	*M. thermoacetica*	−	CO_2_ fixation	Self-assembled (associated with microbe)	Undefined ET pathway	QY (acetate formation): 52 ± 17% (435-485 nm, 5 × 10^13^ photons cm^−2^ s^−1^)	∼1.25 mM acetate produced in 3 days (see reference SI for sample preparation)	Xenon lamp, 75 W	5
CdS NPs	Activity of *D. desulfuricans* biohybrid (under illumination) decreased with Cd^2+^ concentrations above 3 mM	*D. desulfuricans*, *C. freundii*, *S. oneidensis*	−	H_2_ production	Self-assembled (associated with microbe)	Undefined ET pathway	Apparent QY (*D*. *desulfuricans*): 23% (+MV)/4% (no MV)	55 μmol H_2_ produced in 100 h (*D. desulfuricans*); (3 mM Cd^2+^, 100 μmol Cys, 0.5 mM MV, dry cell weight 5.3 mg)	445 nm LED, 0.42 W m^−2^	28
CdS NPs	No cytotoxicity found in presence of CdS (tested under dark)	*R. palustris*	−	N_2_ fixation	Self-assembled (associated with microbe)	Cross-membrane interaction of nanoparticles proposed as transmembrane ET pathway	N/A	∼0.6 μmol C_2_H_4_ produced (h^−1^ mg^−1^ cells) at 18 h (nearly 2-fold compared to natural cells); (see reference for details)	Visible light, 80 W m^−2^	29
CdS NPs	Cell proliferation heavily inhibited by > 100 mM Cd^2+^ without cysteine, and >300 mM Cd^2+^ with 1 mM cysteine (light conditions unspecified)	*E. coli*	−	H_2_ production	Self-assembled (associated with microbe)	ET proposed directly between membrane-embedded CdS particles and intracellular enzymes	Apparent QE: 7.93% (470 nm)/9.59% (620 nm)	400 μmol (additional) H_2_ produced in 3 h (0.3 mM Cd^2+^, 1 mM Cys)	Visible light, 2000 W m^−2^	30
CdS NPs (+/− N-doping)	N/A	*M. barkeri*	N/A	CO_2_ fixation	Self-assembled (associated with microbe)	Transmembrane ET proposed *via* unidentified membrane proteins	QY (CH_4_ production): 39.04 ± 1.34%	0.24 μmol CH_4_ produced (h^−1^); (0.6 mM Ni(0.75%): CdS semiconductor incubated with cells of OD_600_ = ∼0.2)	395 ± 5 nm, 10 W m^−2^	31,32
CdS NPs	Cytotoxicity was not determined, but significant levels of ROS were detected (under illumination)	*S. oneidensis*	−	H_2_ production	Self-assembled (associated with microbe)	Several ET pathways are possible	N/A	362.44 ± 119.69 μmol H_2_ produced (mg^−1^ biohybrid) in 72 h (∼711-fold compared to natural cells); (see reference SI for sample preparation)	Visible light, 1000 W m^−2^	33
CuInS_2_/ZnS	Cell viability unaffected by nanoparticles until at least 36h (under illumination)	*S. oneidensis*	−	H_2_ production	Nanoparticles localised to periplasm (non-specific)	Direct ET from periplasmic nanoparticles to intracellular enzymes	Apparent QE: 15.02% (475 nm)	491.8 ± 26.6 μmol H_2_ produced in 9 h (8.6-fold compared to bare QDs); (28.1% cellular uptake efficiency of 100 mg ml^−1^ Cu QDs, OD_600_ = 1.0)	Visible light, 2750 W m^−2^	34
CdS NPs	Cell viability in 1 mM CdS is ∼70%, which decreased further to ∼40% upon 24h irradiation (2 W cm^−2^)	*S. ovata*	−	CO_2_ fixation	CdS adhere to and encapsulate the bacteria (non-specific)	Transmembrane ET proposed *via* membrane-associated flavoprotein and ferredoxin	QY: 16.8 ± 9%	∼40 mM acetate produced in 4.5 days, rotation of 0.5 day light/dark cycles; (1.0 mM CdS)	400 ± 5 nm LED, 2.0 W m^−2^	35
CdS NPs	N/A	*C. autoethanogenum*	+	CO_2_ fixation	Cell surface associated (non-specific)	Transmembrane ET proposed *via* metal-ion or flavin-binding proteins	N/A	12.1 mM acetate produced in 72 h (3.8-fold compared light-deficient conditions); (0.28 mg L^−1^ CdS)	N/A	36
CdS NRs	*C. necator* withstands higher CdS concentration than other microbes (under illumination)	*C. necator*	−	CO_2_ fixation for bioplastic production	Direct physical contact observed in aggregates (non-specific)	Unclear ET pathway	N/A	1.41 ± 0.13 g L^−1^ PHB produced in 120 h (∼2-fold compared to natural cells); (2 gL^−1^ CdS NR)	LED, 4200 lx	37
g-C_3_N_4_	N/A	*C. necator*	−	CO_2_ fixation for bioplastic production	Direct physical contact observed in aggregates (non-specific)	Unclear ET pathway	QE (NADPH): 8.74 ± 0.70%	6.73 ± 0.45 g L^−1^ PHB produced in 96 h (1.4-fold compared to natural cells); (0.5 g L^−1^ g-C_3_N_4_)	LED, 4200 lx	38
Cu_2_O/RGO nanosheet	N/A	*S. oneidensis*	−	H_2_ production	Nanoparticles and microbes networked *via* RGO (non-specific)	RGO provides ET pathway from nanoparticles to microbe; transmembrane ET *via* membrane cytochromes	N/A	322.0 μmol H_2_ produced (g^−1^ Cu_2_O) in 4 h	Xenon lamp, 300 W	39
Photocatalytic sheet (see text)	N/A	*S. ovata*	−	CO_2_ fixation	Bacteria grow in close contact with nanoparticle clusters (non-specific)	Photocatalytic sheet generates H_2_ (utilised by bacteria) and electrons (transferred into bacteria *via* undefined pathway)	Apparent QY (acetate formation): 21.3% (420 ± 15 nm)	∼9 mM acetate produced in 15 h (OD_600_ = 0.6)	Solar illumination, 1000 W m^−2^	40

Due to a lack of electronic interaction between photosensitisers and microbes, some systems require a redox mediator such as methyl viologen (MV) for electron transfer into bacteria. For example, a biohybrid system has been constructed in which the phototropic bacterium *Rhodopseudomonas palustris* is further photosensitised with conjugated polymers, oligofluorene (OF) and polythiophene (PTP).^22^ The natural H_2_ generation by these bacteria was increased when MV was added to mediate electron transfer from the conjugated polymers to the intracellular enzymes. MV has also been used to shuttle electrons between a TiO_2_ nanoparticle photosensitiser suspended in bacteria culture media with a recombinant strain of *Escherichia coli*. Within the *E. coli* microbe, electrons were transmitted to an exogenously expressed [FeFe]-hydrogenase derived from *Clostridium acetobutylicum* NBRC 13948 to produce H_2_.^23,24^ Photosensitisers Eosin Y and ruthenium-tris-2-2′-bipyridine (Ru(bpy)_3_^2+^) have been coupled to *Shewanella oneidensis* MR-1 as a biocatalyst using MV, driving H_2_ generation along with the reduction of fumarate, pyruvate and CO_2_ to, respectively, succinate, lactate and formate.^25^ An *E. coli* system, expressing [FeFe]-hydrogenases for H_2_ production and using Eosin Y and Ru(bpy)_3_^2+^ as photosensitisers, was enhanced by redox mediators MV and diquat derivative 1,1′-(1,3-propylene)-5,5′-dimethyl-2,2′-bipyridinium (DQ).^26^ Both mediators significantly improved the performance of the system although, due to its more negative reduction potential, a faster reaction rate was achieved using DQ.

Use of mediators is not only unsustainable due to potential toxicity, but also the lack of a defined interface between components. Moreover, as mentioned, when an oxidative half reaction is coupled to the biohybrid system to close the redox cycle in place of an SED, the free redox mediator can cause back reactions and short-circuit the electron for the reductive reaction. Therefore our view is that redox mediators, although a useful research tool, limit optimisation of electronic coupling and thus the longevity of the system.

Biohybrids based on the self-assembly of CdS nanoparticles precipate Cd^2+^ and S^2−^ at the surface of bacteria. The first system to pioneer this method used self-photosensitisation of *Moorella thermoacetica* to drive solar-powered reduction of CO_2_ to acetic acid.^5^ Similar systems constructed with *Desulfovibrio desulfuricans*, *Citrobacter freundii* and *S. oneidensis* MR-1 were used for light-driven H_2_ generation, with *D. desulfuricans*-CdS displaying the highest activity.^28^ Quantum yields of 23% and 4% were obtained for this biohybrid, with and without the addition of MV respectively. The photoheterotrophic bacterium *R. palustris* was coated with CdS nanoparticles for N_2_ fixation.^29^ In this example, CdS has been shown to form clusters that are able to cross the cell membrane, leading to the assumption that this provides a feasible electron transfer pathway within the cell. In the *E. coli*-CdS biohybrid for H_2_ generation,^30^ nanoparticles are localised across the periplasmic space of the *E. coli* membrane. It was proposed that the intracellular surface of the CdS particles could interact with bioactive components and integrate into the hydrogen production pathway. *Methanosarcina barkeri*-CdS biohybrids were able to reduce CO_2_ to methane.^31^ The CH_4_ yield increased by approximately 250% when CdS were doped with Ni.^32^ The introduction of Ni dopant was proposed to suppress the recombination of electron–hole pairs in the biohybrid, leading to enhanced electron transfer. Additionally, Ni induces alterations in the metabolic state of *M. barkeri*, leading to increased expression of various proteins involved in electron transfer, energy conversion, and CO_2_ fixation. It was proposed in this study that photoelectron transfer for CO_2_ reduction occurred through membrane-bound proteins. However, the type of membrane protein was not identified. In a recent study involving *S. oneidensis* MR-1-CdS biohybrids for H_2_ generation, four possible electron transfer pathways were proposed.^33^ Hydrogen could be produced directly by the light-harvesting nanoparticles themselves, by hydrogenase enzymes, by nanoparticle-hydrogenase enzyme hybrids, or by electron transfer from the nanoparticles to intracellular hydrogenases through the outer membrane proteins. The complexity of these systems, and lack of clarity due to multiple possible routes, makes it difficult to ascertain precise electron transfer pathways.

Instead of nanoparticles self-assembled by the bacteria upon addition of starting materials, pre-synthesised nanoparticles (or other nano-scale materials) can be added directly to the bacteria media. For example, CuInS_2_/ZnS quantum dots (QDs) have been added to a *S. oneidensis* strain expressing periplasmic hydrogenases.^34^ The QDs can be taken up into the bacterial periplasm where they interact directly with the hydrogenases for H_2_ production. A *Sporomusa ovata*-CdS hybrid was used to convert CO_2_ to acetic acid. An electron transfer pathway has been proposed^35^ where photoelectrons are simultaneously accepted by the membrane-associated flavoprotein and ferrodoxin before being shuttled to the intracellular space *via* menaquinone. In a *Clostridium autoethanogenum*-CdS hybrid for CO_2_ reduction, it has been proposed that electrons generated from nanoparticles are largely transported to the intracellular matrix *via* metal ion or flavin-binding proteins.^36^ CdS nanorods^37^ and graphitic-carbon nitride (g-C_3_N_4_)^38^ were combined with *Cupriavidus necator* for the production of the bioplastic polyhydroxybutyrate (PHB) from CO_2_ or fructose. It was found that synthesised CdS nanorods with optimised shape and photoactivity improved the performance of the biohybrid system. However, the electron transfer pathway from photosensitiser to bacterium remains unclear. For other systems, more information about transmembrane electron transport is known. In a Cu_2_O/reduced graphene oxide (RGO)-*S. oneidensis* MR-1 biohybrid for H_2_ generation, Cu_2_O acted as the semiconductor for harvesting solar energy.^39^ RGO nanosheets provided a surface to enable efficient photoelectron collection from Cu_2_O and efficient electron distribution to the cells. When metal reducing proteins such as MtrA, MtrB, and MtrC/OmcA (described in detail below) were not expressed, H_2_ generation was significantly inhibited. This suggested that, in this case, these specific outer membrane proteins provided the main electron transfer pathway between the semiconductor and bacterium.

Most existing biohybrid systems are designed for reductive reactions that require SEDs to regenerate the photosensitiser. Systems capable of catalysing an oxidative reaction that do not require a costly SED are rare (see Fig. 1). A pioneering biohybrid system was recently developed which performs H_2_O oxidation and CO_2_ reduction simultaneously, without sacrificial reagents or organic mediators.^40^ In this system, the CO_2_-fixing acetogenic bacterium *S. ovata* is combined with a photocatalytic sheet of nanoparticle clusters (Cr_2_O_3_/Ru–SrTiO_3_:La,Rh|ITO|RuO_2_–BiVO_4_:Mo). Acetate and oxygen are produced by the biohybrid system using only sunlight, CO_2_ and H_2_O. A solar-to-acetate conversion efficiency of 0.7% was achieved at ambient conditions. *S. ovata* used both H_2_ and electrons derived directly from the photocatalytic sheet to reduce CO_2_ to acetate.

The formation of biohybrids using a variety of light-harvesters and many different microbes reflects positively on the robustness of this concept. However, a fundamental disadvantage of some biohybrid systems discussed in this section is the challenge to explain non-specific binding and unclear electron transfer pathways across microbial membranes. Systems for which these pathways are not built in by design require extensive characterisation to understand and improve function, and are at risk of operating in ways other than the desired solar-driven pathway. Our perspective is that this can be mitigated by rationally designing a known electron transfer route to reach the site of catalysis. Microorganisms that naturally contain transmembrane electron transfer machineries provide an elegant design solution for cellular internalisation of electrons, advancing biohybrid systems for which the electron transfer pathway can be more easily defined, understood, and optimised.

## Natural electron transport pathways across the microbial membrane

**Fig. 2:**
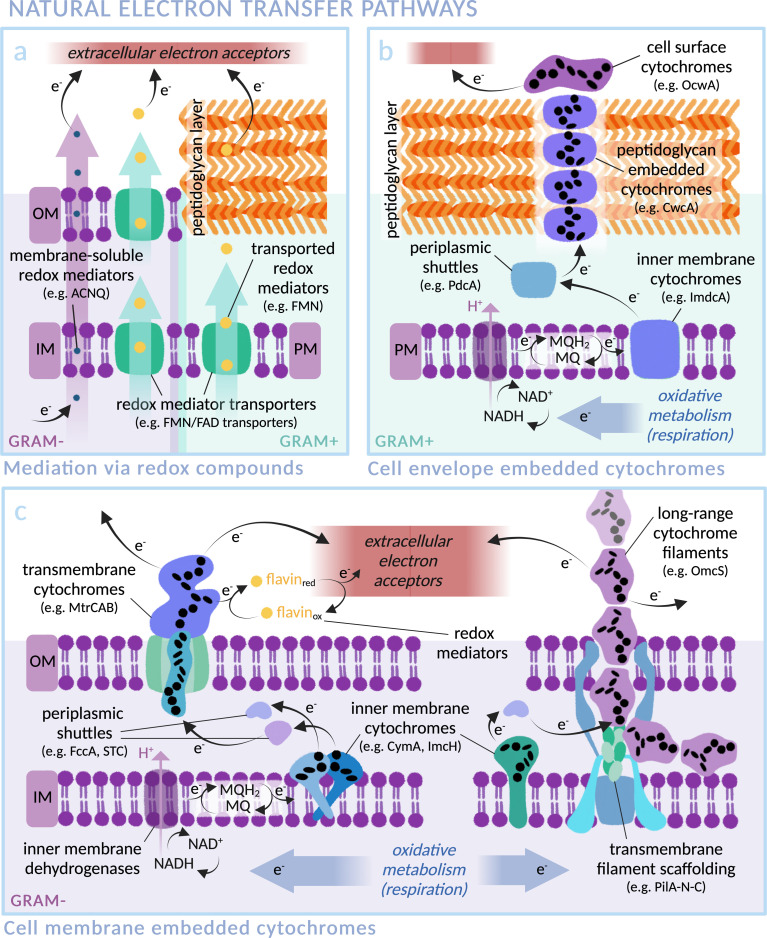
Transmembrane electron transfer in nature: electron transport machineries embedded in (Gram-negative) microbial cell membranes and (Gram-positive) peptidoglycan cell walls. Proteins are arranged according to current literature, with heme groups of select cytochromes indicated as black ellipses. (IM: Inner Membrane, OM: Outer Membrane, PM: Plasma Membrane). (a) Mediated electron transfer *via* redox compounds: membrane soluble compounds (*i.e.* phenazines in *Pseudomonas aeruginosa*,^76,77^ ACNQ in *S. oneidensis*^78^) or *via* transmembrane transporters (*i.e.* FAD/FMN transporters; RibU, FmnA, FmnB and PpIA in Gram-positive *Listeria monocytogenes*,^79,80^ periplasmic FccA and STC in Gram-negative *S. oneidensis*^81^). (b) Representative extracellular electron transfer pathway of the Gram-positive *Thermincola ferriacetica* (*i.e.* surface cytochrome OcwA, peptidoglycan-embedded CwcA filament, periplasmic PdcA and inner membrane ImdcA),^82^ with functional homology to *e.g.* TherJR proteins in *T. potens* and OmhA in carboxydothermus ferrireducens.^72–75,82,83^ (MQ = menaquinone; MQH_2_ = menaquinol) (c) Representative extracellular electron transfer pathway of the Gram-negative *S. oneidensis* (*i.e.* left: outer membrane MtrCAB, periplasmic FccA and STC, inner membrane CymA)^47–49,81,84^ and *G. sulfurreducens* (*i.e.* right: long-range OmcZ/OmcS cytochrome filaments, periplasmic PpcA and inner membrane ImcH).^58,63,85^

In nature, exoelectrogenic microbes can respire using extracellular material as a terminal electron acceptor, while electrotrophs accept electrons from the environment for reductive metabolism.^41,42^ This highly specific functionality has allowed exoelectrogenic bacteria, archaea, microalgae and fungi to occupy extreme environmental niches, for example those rich in minerals and subject to anoxic conditions or fluctuating availability of terminal electron acceptors.^42^ Species across these different kingdoms of life have independently evolved mechanisms and machineries for the exchange of electrons between the intracellular and extracellular environments, commonly involving cross-membrane transport by redox mediators (Fig. 2a) and/or a chain of conductive cytochromes that act as wires through the membrane(s) and peptidoglycan cell wall (Fig. 2b and c). This natural function provides an excellent opportunity to exploit transmembrane electron transfer by coupling exoelectrogens to extracellular electron donors such as electrodes or photosensitisers. Exoelectrogens have been used extensively in systems of microbial electrosynthesis to power reductive metabolism for chemical production, and also within microbial fuel cells (MFCs), which harness electrons released from oxidative catabolism to produce an electrical current.^43,44^ Electron transfer mechanisms in bioelectrochemical systems have been comprehensively reviewed elsewhere.^45,46^ Here, we focus on the mechanistic details of electron transmission within examples of representative species, with an outlook towards integration into biohybrid solar fuel production systems.

The exoelectrogen and facultative anaerobe *S. oneidensis* MR-1 is a versatile species able to respire using a wide range of terminal electron acceptors.^47–49^ With a fully sequenced and annotated genome,^50^ it has been used extensively in MFC studies, leading to a detailed characterisation of the protein machinery between the inner membrane quinone pool and the extracellular space (see Fig. 2c).^43^ The outer membrane spanning Mtr complex, made up of protein components MtrA, MtrB and MtrC, is integral within this pathway. Electrons from the quinone pool are first passed to inner membrane c-type cytochrome CymA. They are then shuttled by periplasmic electron carriers to cytochrome MtrA, a decaheme chain encased within the insulating pore of outer membrane spanning β-barrel MtrB, before emerging on the outside of the cell *via* lipid-anchored decaheme cytochrome MtrC.^51^ MtrC interacts with extracellular electron acceptors and passes electrons laterally to both other MtrC proteins and OmcA, a similar cytochrome in terms of structure and function. A specific arrangement of heme groups was revealed by the crystal structure of MtrC: the shape of a ‘staggered cross’.^52^ Eight heme groups form a roughly linear chain perpendicular to the cell membrane, with the two central hemes also part of an orthogonal tetraheme chain. Rapid electron exchange between these heme groups enables the “nanowire” function.^53,54^ The junction where the heme chains intersect, comprising co-planar and T-shaped heme stacking motifs, allows electrons derived from inside the cell to travel in three possible directions to surface cytochromes or extracellular acceptors with approximately equal efficiency.^55^ Controlling expression levels of MtrCAB has been shown to significantly alter extracellular electron transfer efficiency.^56^

Of key importance for biohybrid development is the possibility to reverse the direction of transmembrane electron flow in *S. oneidensis*; the membrane machinery can be induced to pass electrons from the extracellular space into the cytoplasm.^52^ The energetics of this reversed electron transfer through the MtrCAB pathway were analysed by measuring the electron flux between a counter electrode and a bacteria-coated working electrode, using the cathodic current caused by reduction of fumarate to succinate as a read-out.^57^ Electrons were successfully transferred to periplasmic fumarate reductase FccA. Situated at the surface, MtrC provides a potential entry point for injection of extracellular photoelectrons into the Mtr pathway within a biohybrid system.

In another well studied exoelectrogen *Geobacter sulfurreducens*,^58^ the protein complexes and transmembrane electron transport mechanisms are less well characterised, with two tandem four-gene clusters coding for an assortment of periplasmic and outer-membrane cytochromes thought to be similar in function to the Mtr proteins of *S. oneidensis*.^59,60^ Outer membrane proteins OmcZ, OmcS and OmcE are found in networks of protein filaments extending from the cell surface that facilitate long-range (>10 μm) interaction with extracellular electron acceptors (see Fig. 2c).^61–65^ Similarly to MtrC and OmcA of *S. oneidensis*, these Geobacter proteins have hemes packed in approximately linear chains. OmcE forms thinner filament structures than OmcS and OmcZ, and undergoes a greater number of post-translational modifications, indicating a variation in properties between filament types.^65^ The T-stacked hemes of OmcZ have been shown to pack at a closer distance than those of other Omc proteins in *G. sulfurreducens*, a feature hypothesised to be responsible for the exceptionally high electronic conductivity of this protein (OmcZ filaments have 1000-fold higher conductivity than OmcS). Additionally, a branched arrangement of heme groups is likely integral to the cross-linking function of OmcZ filaments in biofilm formation.^61,62,66^ Despite contrasting amino acid sequences, the physical arrangement of heme groups in an unbranched structure is highly conserved between OmcS and OmcE.^65^ Similarly, conductive filament structures recently noted in two major orders of archaea also exhibit non-homologous protein sequences yet comparable heme arrangements, indicating the convergent evolution of heme structures both within and across multiple species for optimised electron transfer.^67^ In the context of biohybrid design, it could be possible to wire photosensitisers directly to these filaments.

In contrast to *S. oneidensis*, for which only inner membrane cytochrome CymA is linked to extracellular electron transport, *G. sulfurreducens* expresses various different inner membrane cytochromes depending on the types and redox states of available electron acceptors.^68^ ImcH, for example, is required for electron transfer to high redox potential acceptors, but not those below −0.1 V *versus* standard hydrogen electrode (SHE). The opposite response pattern was found for CbcL,^69^ and others such as CbcBA are required for very specific redox potential ranges.^70^ Multiple available electron transfer pathways allow rapid switching in a fluctuating environment. Recently, the formation of intracytoplasmic membrane structures has been discovered.^71^ It is proposed that these extra inner membrane folds provide a larger surface area for electron transfer proteins under low energy conditions, in order to increase the quantity of electrons able to be transmitted from the cytoplasm. This native versatility makes *G. sulfurreducens* a highly promising candidate for scalable biohybrid systems. Multi-heme membrane cytochromes are also found in several Gram-positive bacteria, such as *Thermincola potens*, where a stacked chain of electron conduit proteins are hypothesised to transfer electrons through the cell wall (see Fig. 2b).^72–75^ Many exoelectrogenic archaea are also reliant on cytochromes for transmembrane electron transfer although, intriguingly, there are examples of archaea that are able to pass electrons to other microbes but do not express multi-heme proteins, indicating alternative electron transfer pathways in nature.^42^ Despite the gaps in knowledge that remain for many exoelectrogenic species, the certainty that there exists a natural transmembrane electron transfer pathway to be harnessed makes this class of microbes a promising choice for biohybrid systems.

Though the mechanistic details of electron transfer in exoelectrogens are continually emerging, injecting electrons into the cell is not so simple as directly reversing the natural pathway; some research has shown that there may be routes which bypass the Mtr pathway.^86^ Engineering exoelectrogens to enhance transmembrane electron transport could improve future systems.^87^ As well as further characterisation of these machineries,^57,88^ the interface between photosensitiser and microbe should become a major complimentary research focus. Direct electron injection is a crucial and under-researched stage of electron transfer in biohybrid design. Rational design of this interface is required, taking into account both physical attachment and electron transfer between components.

## Rational design of the photosensitiser-protein interface

Many current biohybrid systems are limited by a lack of molecular design and control at the photosensitiser–microbe interface.^17^ Here, we highlight a range of research that would be valuable to explore when designing this essential interface: hybrid proteins. These are systems in which a photosensitiser has been specifically bound to a protein, often an isolated redox enzyme, to create a direct and defined path of electron transfer.^16,89^ Fast electron transfer is necessary for the optimisation of biohybrid systems. This needs precise engineering at the molecular level; according to the Moser–Dutton ruler, a distance of ≤14 Å is required between the photosensitiser and the distal redox co-factor of the enzyme.^90^

[NiFeSe]-hydrogenase enzymes^91,92^ from *Desulfomicrobium baculatum*, among others, have been photosensitised using ruthenium-dye-sensitised titanium oxide nanoparticles.^93^ Attachment of TiO_2_ nanoparticles to hydrogenase enzymes was inspired by the stable electrochemistry observed when the enzyme was adsorbed onto a TiO_2_-ITO electrode in comparison to graphite electrodes.^94^ Coupling TiO_2_ nanoparticles to a ruthenium photosensitiser expanded the range of photoabsorption into the visible range, with TiO_2_ extending the lifetime of the charge separated state and acting as an attachment surface for the hydrogenase. There are several systems which use the ability of TiO_2_ to adsorb enzymes, including a CO_2_-reducing enzyme (CODH) from *Carboxydothermus hydrogenoformans*^95^ and formate dehydrogenase (FDH) from *Desulfovibrio vulgaris*.^96^ TiO_2_-adsorbed CODH has been coupled to light absorbing Ag nanoclusters stabilised with polymethacrylic acid.^97^ In addition to Ru-sensitised TiO_2_, biohybrid systems are established for polymeric carbon nitride (CN_*X*_), CN_*X*_-TiO_2_ and Eosin Y as photosensitisers.^98–100^ A [NiFeSe]-hydrogenase system photosensitised with CN_*X*_, despite a weaker photosensitiser-enzyme interaction than for Ru-sensitised TiO_2_, demonstrated much higher stability and remained active for 2 days compared to 8 hours.^94,98^ The interaction was enhanced by adding TiO_2_ as a surface for adsorption (CN_*X*_-TiO_2_).^99^ However, TiO_2_ systems have the disadvantage of generating toxic reactive oxygen species (ROS) in the presence of O_2_. To overcome this limitation, Eosin Y was introduced as a photosensitiser. This organic dye actively protects the enzyme by reacting with O_2_ to create a pseudo-anaerobic environment under air. Even in atmospheric conditions, the light-driven hydrogenase was able to maintain ∼11% of the photoactivity measured under anaerobic conditions.^100^ In an alternative approach, CN_*X*_ has been recently demonstrated to exhibit a strong electrostatic interaction with an [FeFe]-hydrogenase from *Clostridium pasteurianum*^101^ without the need for TiO_2_.

Light-harvesting nanoparticles such as carbon dots (CDs) have been functionalised with surface groups to engineer the nature of their interaction with an enzyme. Likely involving hydrogen bonding, FDH was found to bind CDs functionalised with either positive (-NHMe_2_^+^) or negative (–COO^−^) groups in equivalent amounts.^102^ However, the CD surface chemistry affects their localisation on the protein surface and thereby (presumably) the kinetics of photoelectron transfer: CDs with positive surface groups were able to drive CO_2_ reduction to formate in the presence of a sacrificial electron donor, while CDs with negative surface groups were not. This demonstrates how integral the positioning of the photosensitiser component is for successful electron transfer in biohybrid systems. Another study investigated the interaction of functionalised CDs with FccA and [NiFeSe]-hydrogenase enzymes and drew a similar conclusion.^103^ In a different example, the electrostatic interaction between CDs and an [FeFe]-hydrogenase enzyme was disrupted by the addition of ethylenediaminetetraacetic acid (EDTA) as an SED, with hydrogen evolution after 1 hour around 4.4-fold higher when replaced by a different SED: triethanolamine (TEOA).^104^

The nature of electrostatic self-assembly between QDs and enzymes has been analysed in detail for CdS and CdTe nanoparticles coupled to hydrogenase enzymes.^105^ Functionalisation of these nanoparticles with a self-assembled monolayer of 3-mercaptopropionic acid (3-MPA) allowed solvation in an aqueous environment and, due to the negative charge of exposed carboxylate groups, facilitated interaction with positively charged areas of the protein surface. A *C. acetobutylicum* [Fe–Fe]-hydrogenase, with surface-localised [FeS] clusters accessible for direct electron transfer, was adsorbed to 3-MPA-functionalised CdS nanocrystals.^106^ The rate of catalysis was shown to increase with hydrogenase only up to a certain enzyme:CdS ratio before declining, an effect attributed to back electron transfer due to steric hindrance of the hydrogenase crowding. An optimised ratio of 0.67 : 1 for hydrogenase : CdS achieved ∼20% quantum yield for H_2_ production. This value remained constant over a range of light intensities, indicating that the limiting step was not the rate of electron transfer or catalysis, but rather the rate of photoelectron generation. The quantum yield could thus be improved in this example by increasing the absorbance range of the photosensitiser component, but the electron transfer interface itself is considered highly effective. 3-MPA-functionalised CdS nanorods/nanocrystals and other QDs have also been adsorbed to an *Azotobacter vinelandii* nitrogenase [Mo–Fe] catalytic centre.^107,108^ The production yield when using CdS nanorods as a photosensitiser far outcompeted that of Ru-based compounds for this example, indicating the extent to which the photosensitiser component can affect the functionality of a catalyst.^109^ As described for hydrogenase systems, the nature of physical attachment between functionalised CdS photosensitisers and the protein is attributed to self-assembly *via* surface electrostatics.^106,110^ Physical binding of CdS to the enzyme in solution was demonstrated using dynamic light scattering (DLS), with the hybrid exhibiting a significantly higher average hydrodynamic diameter than either individual component. Characterisation of the electron transfer interface was investigated extensively in terms of quantum efficiencies and kinetics, with formation of a single complex concluded to be integral for efficient electron transfer and product formation.^111–113^ 3-MPA-coated CdS nanorods have also been coupled to an oxidoreductase enzyme from *Magnetococcus marinus* MC-1.^114^

H_2_ production was achieved in a similar electrostatically conjugated hydrogenase system, with 3-MPA-functionalised CdTe nanocrystals as the light-harvesting component.^110^ The CdTe-hydrogenase hybrid was confirmed to form a stable single complex using Native Polyacrylamide Gel Electrophoresis (PAGE). With this technique, CdTe nanocrystals were clearly seen to form a fluorescent hybrid species with hydrogenase on the gel. The kinetics of adsorption were analysed: despite being weaker than a true covalent bond, the interaction was concluded to form a stable complex. Electron transfer between components was also investigated. Contrary to expectations, it was found that mixing at a lower hydrogenase : CdTe ratio resulted in much higher electron transfer efficiency and light-driven H_2_ production rate. These observations indicate that in this case, a lower ratio of enzyme per nanocrystal made for a more optimal system, a result attributed to high enzyme coverage forcing hydrogenases into an orientation less effective for electron transfer.^110^ A study involving uncoated CdS nanomaterials coupled to CODH additionally highlights the effect nanoparticle shape and size can have on catalytic activity.^115^ Due to the importance of this interface for electron transfer efficiency, the position, size and quantity of light-harvesters per microbe are important factors to optimise.

Closing the redox cycle is an important challenge to address. Very few examples of this have been achieved across any system of biohybrid photocatalysis, with water oxidation an obvious target when considering fuel production on a global scale.^40^ However, other substrate candidates aside from water are feasible for oxidation in a scaled-up system, for instance biomass (lignocellulose),^116^ which is already widely available and circular, or plastic^117^ (which will likewise become circular).^118,119^ Recently, the reductive chemistry of a nanoparticle-enzyme biohybrid was successfully coupled to a value-adding oxidative reaction: TiO_2_-adsorbed FDH enzymes catalysed reduction of CO_2_ to formate, while the TiO_2_ photosensitiser was regenerated through oxidation of glucose to arabinose and formate.^116^ This example neatly demonstrates the ability to design a system which couples two redox reactions and generates multiple desirable products, eliminating the need for a costly and thus uneconomical SED. Compartmentalised photocatalysis becomes highly important in these systems to eliminate back-reactions.

**Fig. 3:**
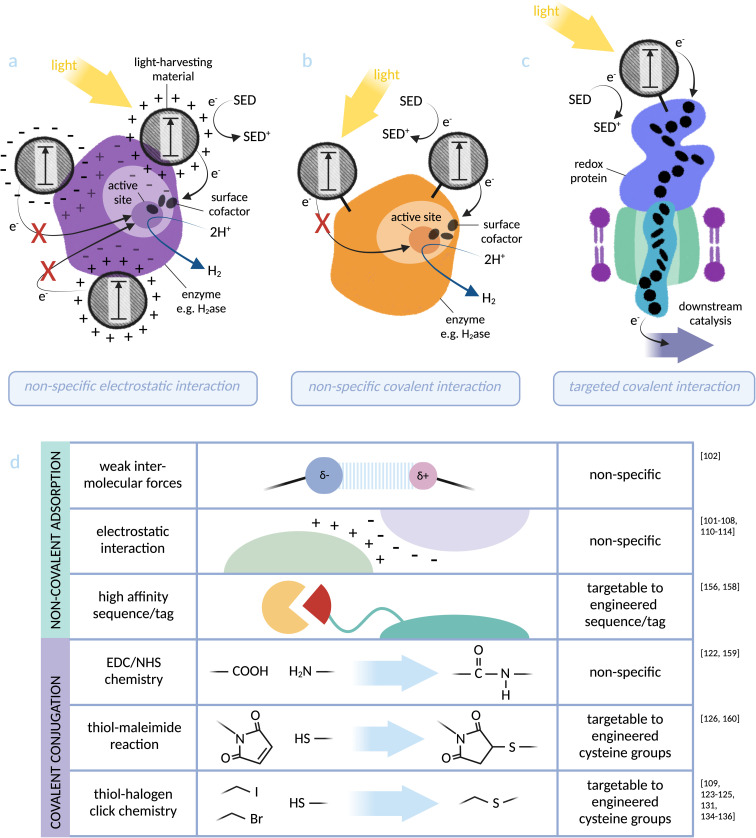
Photosensitiser-protein hybrid interfaces: (a) non-specific electrostatic interaction between a light-harvesting material and an enzyme (based on [NiFeSe]-hydrogenase):^103^ distance dependency of electron transfer to the catalytic centre makes catalytic activity directly dependent on the location of the nanoparticle, which can be investigated but not controlled using this binding method. (b) Non-specific covalent binding of a light-harvesting material to lysine residues at an enzyme surface (based on [NiFe]-hydrogenase):^122^ proximity to the active site can again be investigated but not controlled *via* this method. (c) Specific covalent conjugation between a light-harvesting material and a transmembrane cytochrome (based on MtrCAB from *S. oneidensis*):^126^ binding directly *via* an engineered cysteine residue allows targeting to an optimal site for electron injection into the redox protein. (d) Summary of the physical photosensitiser-protein binding methods discussed in this perspective. Note: some studies^91–93,95–99,115,116,127,128,130^ do not distinguish between weak intermolecular forces (such as van der Waals and hydrogen bonding) and stronger electrostatic interactions and these have not been included in the figure.

While the examples above are successful electron transfer interfaces, coupling methods that rely on non-specific adsorption are not inherently transferable to whole-cell biohybrids due to the complexity of the microbial surface.^120,121^ An approach where the photosensitiser is covalently tethered to the protein might solve this issue, and has two major advantages: (a) the method can be more easily applied to any system with chemically adjustable components (*e.g.* chemical dyes and functionalised nanoparticles), and (b) it is possible to target photosensitisers to a specific site, thus allowing optimisation of the electron transfer interface. CdS/CdSe quantum rods coated with carboxylic acid-containing ligands have been covalently bound to a [NiFe]-hydrogenase from *Aquifex aeolicus via* amide bond formation with lysine residues.^122^ Biohybrid complex formation was verified by both colocalisation of nanoparticle and enzyme on native PAGE and peptide mass fingerprint. Expanding this method to bind a targeted site on the enzyme would allow further optimisation of the electron transfer interface (see Fig. 3). A cysteine-binding Ru-based photosensitiser has been covalently bound to an engineered cysteine residue in cytochrome P450 BM3 from *Bacillus megaterium*, driving integration of atmospheric O_2_ into hydrocarbons within the heme-based catalytic centre of the enzyme.^123–125^ Targeting the Ru electron donor site to an optimised location for electron transfer required comparing cysteine mutants at several positions on the enzyme, each of which significantly affected catalytic rate. This highlights the sensitivity of the abiotic–biotic interface on electron transfer, and reiterates the importance of designing and investigating an optimal physical interaction for efficient electron transfer.

## Coupling to membrane cytochromes of exoelectrogenic microbes

Building on the concept of interfacing redox enzymes with photosensitisers, a small number of studies have demonstrated binding light-harvesting materials directly to the membrane cytochromes of exoelectrogenic bacteria. While these membrane proteins are not enzymes themselves, the concept of creating an electron transfer interface from light absorber to redox protein for downstream catalysis is translatable between the two research areas. Coupling transmembrane cytochromes to light-harvesters and achieving electron transfer provides a convincing proof-of-principle for the ultimate application of photosensitising microbes.

As discussed, exoelectrogenic bacteria have the ability to transmit electrons between intracellular and extracellular environments.^48^ The transmembrane protein complex MtrCAB from *S. oneidensis* MR-1 provides the point of entry for extracellular electrons to access intracellular electron transfer pathways.^43,52^ A selection of light-harvesting nanoparticles were tested for adsorption with MtrC, the outward-facing protein subunit, assembled as a monolayer on electrode surfaces: RuP–TiO_2_–COO^−^ (negatively charged), RuP–TiO_2_–NH_3_^+^ (positively charged), CdS–COO^–^ and CdS–N(CH_3_)_2_H^+^.^127,128^ It was found that binding of MtrC was highest and most rigid for RuP–TiO_2_–COO^−^. The photocurrent was linearly dependent on light intensity, indicating that the electron transfer interface was not limiting. However, variation in binding properties between these different nanoparticles demonstrates the difficulty in predicting interactions between components. Non-specific adsorption (see Fig. 3) is inherently difficult to predict or control. These results reinforce the perspective that binding methods with built-in specificity, such as targeted covalent binding, are essential for guaranteeing an efficient electron transfer interface.

A recent study from our group has addressed this challenge by rationally designing the covalent conjugation of MtrC with light-harvesting CD nanoparticles.^126,129^ Previously, we coupled MtrC to Ru-based photosensitisers (a) adsorbed on TiO_2_ nanoparticles^130^ and (b) *via* site-specific covalent labelling.^131^ The latter achieved ultrafast electron transfer on the ps timescale. CDs were introduced to this biohybrid design as a comparably sustainable source of light-harvester. The surface groups of CDs are amenable to chemical modification, and they have been previously demonstrated to pass electrons to the MtrCAB complex when mixed free in solution.^132^ Covalent binding was achieved through functionalisation of carboxylic acid residues at the CD surface with maleimide groups, followed by reaction with an engineered cysteine residue of MtrC. The covalent conjugation of photosensitiser-protein components was verified on native PAGE, where the conjugated protein was visualised to carry CD fluorescence and migrate as a wider, more diffuse band (indicating addition of the heterogeneously sized CD particles). Electron (or energy) transfer was shown to occur between components: the fluorescence lifetime of the CD became shorter when interfaced with MtrC. This chemical modification of MtrC demonstrates the use of sustainably produced, low-cost components to create a system that could in theory be translated into a whole-cell biohybrid system using *S. oneidensis* MR-1 for solar fuel production.^132^

PpcA, a periplasmic cytochrome from exoelectrogen *G. sulfurreducens*,^133^ has been conjugated to cysteine-binding Ru-based photosensitisers for the same purpose. In this study, site-directed mutagenesis was used to introduce non-native cysteine residues to PpcA as a model for targeted conjugation.^134^ The aim was to form chiral linkages with photosensitiser components, allowing sterically specific conformations that mimic the physically constrained photosystem structures in nature. Structural aspects of the conjugates were analysed alongside rates of photo-induced electron transfer (PET). With PET rates of 6 ps, 130 ps and 35 ns for 3 different mutants, a clear preference for location of electron injection was demonstrated. It was also shown that certain mutants favoured one enantiomer of the chiral photosensitisers, indicating different environmental constraints at each position. A further study validated the conclusion that the tertiary structure of the conjugate directly controls efficiency of electron transfer, this time focussing on comparing cysteine positions introduced directly into heme binding motifs.^135^ Similar conclusions were reached in a study of Ru-dye sensitised STC, a periplasmic cytochrome from *S. oneidensis* MR-1.^136^ These detailed analyses of geometries, interactions and flexibility at the site of conjugation are invaluable for rational design of biohybrids such as these going forwards. While PpcA and STC are less likely to be candidates for translating into a microbial biohybrid system, being located in the periplasm, these proof-of-principle studies lay the foundations for fundamental design principles: precise structural control of the point of electron injection from light-harvesting materials to the heme chains of cytochrome proteins.

The examples discussed above have been chosen to highlight advantages and disadvantages of various protein conjugation methods for translation into whole-cell biohybrid systems. Interfacing photosensitisers with redox proteins requires nuance and rational design. An optimum conjugation method will depend greatly on the nature of the components and the desired interaction. To achieve competitive electron transfer efficiencies in a biohybrid system, an evidence-based approach to directing the site of electron exchange between components is required. While it could be argued that there currently exists too little characterisation for strategic design in this manner, we propose that research should continue to pursue a deeper understanding of prospective biohybrid proteins and their interactions with photosensitisers to accelerate the innovation of successful system designs in the future.

## Toxicity of non-biological components

The toxicity of synthetic light-harvesters on bacteria, and their effect on the environment when applied at a large scale, both play a crucial role in ensuring longevity, sustainability and feasibility of biohybrid systems. This aspect has received limited attention in other reviews on biohybrid systems and is not consistently mentioned in studies. As discussed previously, many different light-harvesters have been employed in biohybrid systems (Table 1). Toxicity is dependent on the type of light-harvester, the concentration, the species of microorganism, and conditions such as light intensity. In this perspective, we aim to provide a more thorough discussion of this critical factor (see Table 1 for a summary of known photosensitiser toxicities under specific conditions, including light/dark, for the examples of solar-driven biohybrids discussed).

Biohybrid systems using water soluble dyes as a photosensitiser such as Eosin Y and ruthenium complexes often use a redox mediator to shuttle the electron into the bacteria.^22–25,100^ MV has been identified as toxic to microorganisms. Alongside other disadvantages previously discussed, this factor makes it unsuitable for long term use. Additionally, organic dye Eosin Y has been found to be harmful for both microorganisms and human health, exhibiting increased production of ROS under visible light illumination.^137,138^ Ruthenium photosensitisers are specifically used for anticancer therapy and as antimicrobial agents due to their cytotoxicity.^139,140^ Metal-based photosensitisers in general can often result in the incidental dissolution and accumulation of toxic metal ions. The toxicity of these components in the context of specific microbial biohybrid systems, however, is not well researched.

QDs are semiconducting nanoparticles that have unique photophysical properties. The nature of the primary material, the size and shape of the nanoparticles and their specific surface functionalisation are all factors that play an essential role in their potential toxicity.^141^ However, the precise toxicity of nanomaterials to microorganisms and the natural environment is a serious concern that remains largely undetermined, despite extensive research in this area.^142,143^ Cadmium-containing QDs such as CdS and CdTe are most commonly used and have been applied in biohybrid systems involving both redox enzymes and bacteria.^5,33,106,107,110^ Cd^2+^ exhibits high toxicity to a range of microorganisms including bacteria, algae and fungi.^144^ CdS nanoparticles can either be directly added to the bacteria, or formed *via* bio-precipitation on the bacteria in the presence of cysteine. Cell proliferation for *E. coli* was shown to be heavily inhibited with a Cd^2+^ concentration higher than 0.1 mM in the absence of cysteine.^30^ On addition of cysteine, cell growth significantly increased for Cd^2+^ concentrations up to 0.4 mM. However, despite this reduction in toxicity, cysteine addition to biohybrid systems has disadvantages: due to relying on excess thiol for stabilisation, there is no obvious way to couple bio-precipitated CdS systems to a meaningful oxidative reaction, and additionally cysteine can itself be metabolised by some microbial systems.^145^

Various QDs added directly in solution (CdTe/CdS/TGA, CdTe/CdS/cysteamine, CdTe and CuInS2/ZnS/PMAL) have been evaluated for their nanotoxicology with *S. oneidensis* MR-1, using a colony-forming units method and a fluorescence viability assay.^146^ CdTe and CuInS_2_/ZnS/PMAL QDs displayed no toxicity, but CdTe/CdS/TGA, CdTe/CdS/cysteamine QDs significantly inhibited the bacteria growth. In another study, seven different core–shell QDs were tested for toxicity to non-photosynthetic bacteria (*A. vinelandii* and *C. necator*) for the purpose of solar fuels and chemicals generation.^147^ The surfaces of the QDs were each capped with ligands that affected surface charge: negatively charged MPA, positively charged cysteamine (CA), and zwitterionic cysteine. The Cys-capped QDs showed no inhibition on bacteria growth. However, MPA-capped QDs impaired growth moderately, and CA-capped QDs strongly, clearly illustrating how surface chemistry affects the toxicity of nanoparticles. It has also been demonstrated that activity in *D. desulfuricans*-CdS biohybrids decreased when cadmium concentrations surpassed 3 mM, likely due to toxicity.^28^ In the *S. ovata*-CdS hybrid system, a colony-forming unit assay showed that cell viability in the CdS-containing biohybrid was decreased compared to that of cells without CdS, further indicating toxicity of Cd-based photosensitisers.^35^ Toxicity of CdS was tested on the microorganisms *C. necator*, *Saccharomyces cerevisiae*, *Gluconacetobacter xylinus*, and *Anabaena* sp. PCC7120 using different concentrations of CdS nanorods, with toxicity observed for all except *C. necator*.^37^ Nanoparticles can also have toxic effects on the metabolism of different microbes, including protein expression. An example is *Xanthobacter autotrophicus* in which 727 proteins were upregulated and 53 downregulated upon addition of CdTe nanoparticles.^148^ Proteomic and metabolic analyses show that significant alterations occurred for electron transport chain and redox signalling pathways. Expression of N_2_-fixation related proteins such as nitrogenases was increased, while expression of ATP synthase and overall ATP concentration was lowered. Interaction between *S. oneidensis* MR-1 and several nanoparticles including CdS has been shown to increase biofilm formation.^149^ Addition of TiO_2_ has been shown to increase riboflavin secretion.^150^ In conclusion, it is difficult to predict the toxic or metabolic effects of different light-harvesting materials on different organisms.

Gold nanoclusters (AuNCs), considered to be a less toxic material than cadmium-containing QDs, were used as photosensitisers (accumulating intracellularly) for the acetogenic bacterium *M. thermoacetica.*^151^ Both with and without the presence of AuNC, the *M. thermoacetica* system could maintain a relatively high proliferation rate and cell viability until the fourth day, demonstrating biocompatibility of these AuNCs. Recently, carbon-based nanomaterials such as CN_*X*_ and CDs have been applied to biohybrid systems for photobiocatalysis.^98,99,103,104,152^ CDs are generally considered to be environmentally benign. Biocompatibility with *S. oneidensis* MR-1 was evaluated by assessing the viability of bacteria incubated with CDs. Cell viability was found to be maintained >96% after 94 h, confirming the high compatibility and low cytotoxicity of the CDs for this bacterium. They have, however, been implicated in increased intracellular signaling.^153^ Organic semiconductors such as perylene diimide derivative (PDI) and poly(fluorene-*co*-phenylene) (PFP) are also considered non-toxic as a photosensitiser for biohybrid semi-artificial photosystems.^154^

In comparison to organic dyes, nanoparticles offer greater potential for adjustment of both their band gap and surface chemistry. Their high stability and durability are advantageous. However, the impact of nanoparticles both on the environment and the bacteria remains uncertain, with toxicity varying depending on the species of microbe involved. To create a more sustainable system, it is imperative to focus on development of benign photosensitisers such as gold nanoclusters, organic semiconductors, and carbon-based nanomaterials which exhibit biocompatibility and lower toxicity.

## Outlook

The aim of this perspective is to pitch a forward-looking “road-map” for the future of whole-cell solar biohybrids, highlighting a range of research areas that can be explored for solutions to limitations of current systems. Exploiting microbes with natural pathways of transmembrane electron transfer provides a characterisable route for internalisation of photoelectrons, and strategies for conjugating photosensitisers to enzymes can be explored for transferable electron transfer pathways that facilitate a controllable and optimisable interface. Rather than providing a conclusive set of studies, we propose that the field continues to examine research across these areas for inspiration, harnessing new materials, components and conjugation methods from a wide range of studies for application in the whole-cell biohybrid vision.

The main focus has been to highlight methodology used to anchor two redox components together in a such a way that electron transfer is achieved. However, it is also possible to examine protein conjugation methods for purposes such as energy transfer. While electron transfer requires a distance of ≤14 Å between redox centres (according to the Moser–Dutton ruler), energy transfer requires a distance of a 100–500 Å. Physical conjugation methods can nonetheless be explored. A light-harvesting reaction centre (RC) from *Rhodobacter sphaeroides* has been hybridised with CdTe QDs, enabling energy transfer from QDs to occur in a similar way to antennae pigments in nature.^155–157^ CdTe QDs were bound with high affinity to an engineered His-tag with a modifiable linker sequence at the RC surface.^156,158^ Methods to conjugate light-absorbing organic dyes and fluorophores have been established that exploit the presence of available cysteine and lysine residues on the RC. A synthetic aryleneethynylene fluorophore, designed to act as an antenna pigment, was covalently bound to lysine residues *via* a succinimidyl ester group at a ratio of 4.1 ± 0.3 fluorophores per protein.^159^ In another study, fluorescent dyes functionalised with maleimide groups were targeted to engineered cysteine residues.^160^ These types of studies highlight further translatable conjugation methods for targeting light-harvesters to specific locations on a protein.

In a different approach, Texas Red (TR)-modified phospholipid has been used for energy transfer to light-harvesting complex II (LHCII) from spinach.^161,162^ In this system, the light-havester is not coupled to a protein, but to the membrane. Further studies expand the concept to include dye-conjugated phospholipids ATTO647N-DOPE and Cyanine-7-DOPE, and lipophilic dialkylcarbocyanine dyes DiI and DiR.^163^ This type of interaction demonstrates the possibility of designing an energy (or electron) transfer interface between membrane-anchored components *via* proximity rather than direct physical conjugation. However, organic dyes do not absorb a broad spectrum of light, and are less durable than alternative photosensitisers such as semi-conducting nanoparticles. COOH-coated semi-conducting graphitic CDs have been modified with a phospholipid.^164^ Chemically functionalising membrane components with nanoparticles such as this could be highly applicable to the overall biohybrid concept.

An alternative to labelling natural membrane components is to create synthetic membrane-insertable photosensitisers such as amphipathic fluorescent nanoparticles. Lipophilic CDs have been developed that undergo self-assembly into structures similar to liposomes (CDsomes).^165^ They have been used for selective labelling of membrane structures in live mammalian cells, demonstrating high biocompatibility and negligible cytotoxicity, along with the key ability to insert into natural cellular membrane environments. Due to their ability to photoluminesce, these CDs have additionally been investigated for use in photocatalysis.^166^ At 365 nm, CDsomes exhibit a fluorescent on-state, and at 530 nm a non-fluorescent off-state. Using this photo-switchability, it was found that in the on-state catalysis of O_2_ to H_2_O_2_ was driven, and in the off-state ROS were generated. The principle of using a synthetic membrane-embedded CD for photochemistry is fascinating for further research. In another study, a synthetic transmembrane photosystem I mimic has been developed that, on excitation by visible light, is able to pass electrons to an organic dye.^167^ The oligofluorene chromophore structure spans the width of an average phospholipid bilayer, situating itself in a perpendicular orientation that mimics natural transmembrane structures. Photons are channelled to an energy acceptor, generating photoelectrons that can be transferred to acceptors at the membrane surface. Energy transfer between the synthetic light-harvester and organic dye Eosin Y was demonstrated in liposomes by measuring fluorescence emission quenching. Another system uses a perylene diamine chromophore that imbeds in liposomal membranes.^168^ Use of liposomes as a platform for energy transfer, electron transfer and chemical conversion is a branch of its own within the field of developing bio-based systems for solar fuel generation.^169,170^ Within the context of designing whole-cell systems, the relevance of synthetic membrane components such as these can only be judged by their ability to insert into the biological membranes of microbes and interact with native components. For many of these synthetic chromophores, this remains to be seen.

In conclusion, we propose that a broad range of research be used to inform the strategic design of biohybrid systems. The route of a photoelectron, from generation to intracellular catalysis, can be broken down into two main optimisable stages: the interface between photosensitiser and microbe, and internalisation of the electron by the microbe. Delving into the fundamental interactions between photosensitiser and redox protein components will reduce the unknowns when translated into larger, more complex systems. Using known methods for directly tethering key biohybrid components can allow control and optimisation of their interaction. In many cases, there is not yet enough understanding of how the building blocks interact: it is therefore of high importance to continue investigating and engineering these materials and interfaces, and to ultimately use this accumulated knowledge to establish blueprints for the future rational design of solar-driven biohybrids for fuel production.

## Author contributions

Imogen L. Bishara Robertson: conceptualization, writing – original draft, writing – review & editing, and visualization; Huijie Zhang: conceptualization, writing – original draft, writing – review & editing, and visualization; Erwin Reisner: conceptualization, writing – review & editing, supervision, project administration and funding acquisition; Julea N. Butt: conceptualization, writing – review & editing, supervision, project administration, and funding acquisition; Lars J. C. Jeuken: conceptualization, writing – review & editing, supervision, project administration, and funding acquisition.

## Conflicts of interest

There are no conflicts to declare.
